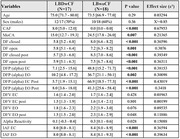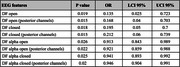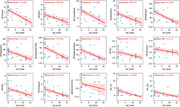# Resting‐State EEG Features of Cognitive Fluctuations in Patients with Lewy Body Dementia

**DOI:** 10.1002/alz.091756

**Published:** 2025-01-09

**Authors:** Ahmed Negida, Sarah Lageman, Nitai Mukhopadhyay, Matthew J Barrett

**Affiliations:** ^1^ Virginia Commonwealth University, Richmond, VA USA

## Abstract

**Background:**

Lewy body dementia (LBD) is characterized by fluctuations in arousal and alertness, i.e., cognitive fluctuations (CF). Although CF significantly impacts quality of life, its neurophysiological basis remains poorly understood. This study's objective was to identify specific EEG features associated with cognitive fluctuations in patients with LBD.

**Method:**

We conducted a cross‐sectional study of 35 patients prospectively enrolled through the outpatient clinics of the Department of Neurology at Virginia Commonwealth University. Based on the Clinician Assessment of Fluctuations, participants with Parkinson’s disease, Parkinson disease with dementia, and dementia with Lewy bodies were categorized as Lewy body disease without CF (LBwoCF) and Lewy body dementia with CF (LBDwCF). All patients underwent resting‐state EEG recording with eyes closed and eyes open for 3 minutes each. EEG data were preprocessed and cleaned, and the following features were extracted: dominant frequency (DF), dominant frequency variability (DFV), dominant frequency prevalence (DFP) within the alpha band (8 to 13 Hz), individual alpha peak frequency (IAF), and alpha reactivity. Kruskal‐Wallis tests and logistic regression models were used to evaluate the relationship between EEG features and group (LBDwCF vs. LBwoCF).

**Result:**

We analyzed EEG features for 17 LBDwCF and 18 LBwoCF. For both EEG conditions, eyes open and eyes closed, the LBDwCF group had significantly lower DF, posterior DF, DFP (alpha), and IAF but not alpha reactivity or DFV, compared to LBwoCF (all P<0.003, Table 1). The EEG feature with the largest effect size (ε²=0.43) was DFP within the alpha band measured from the posterior electrodes in the eyes closed condition. These EEG features, except for IAF, remained significant predictors of group (LBDwCF vs. LBwoCF, all P<0.03, Table 2) in logistic regression models adjusting for age and Montreal Cognitive Assessment (MoCA) score.

**Conclusion:**

Among patients with LBD and PD, resting‐state EEG features were associated with CF. These features were significant predictors of CF even after adjustment for MoCA scores and age. The development of an EEG‐based biomarker of cognitive fluctuations may improve diagnosis of this clinical feature and thus diagnosis of LBD.